# Complete mitochondrial genome of *Francolinus pintadeanus* (Galliformes: Phasianidae)

**DOI:** 10.1080/23802359.2016.1197059

**Published:** 2016-08-30

**Authors:** Xue-Juan Li, Li-Liang Lin

**Affiliations:** College of Life Sciences, Shaanxi Normal University, Xi’an, China

**Keywords:** *Francolinus pintadeanus*, mitochondrial genome, phylogenetic analysis

## Abstract

The complete mitochondrial genome (mitogenome) of *Francolinus pintadeanus*, consisting of 16,693 bp, was determined and analyzed. It displayed as typical genome organization as other Galliformes mitogenomes: 13 protein-coding genes, two ribosomal RNA genes, 22 transfer RNA genes and one control region. The phylogenetic relationships of 25 Phasianidae species and three Odontophoridae species as outgroup using maximum likelihood and Bayesian inference methods based on a concatenated dataset from mitogenomes were analyzed. The results reveal that *F. pintadeanus* had a close relationship with *Gallus gallus gallus*/*Bambusicola thoracica*, then this clade formed a sister group with *Pavo muticus*/*Argusianus argus*.

The Chinese Francolin (*Francolinus pintadeanus*) belongs to Galliformes, Phasianidae. *F. pintadeanus* is distributed in Cambodia, China, India, Laos, Myanmar, Philippines, Thailand and Vietnam. Its natural habitats are subtropical or tropical dry forests and subtropical or tropical moist lowland forests. Hall (1963) regarded all spurfowls and francolins as one genus (*Francolinus*) (Phasianidae, Galliformes), and divided the genus *Francolinus* into eight groups based on morphological and ecological data, with seven distributed in Africa and one in Asia. The phylogenetic relationships of *Gallus*, *Bambusicola* and *Francolinus* were relatively robust in many studies (Shen et al. 2010; Meiklejohn et al. 2014). However, the sister group of the clade (*Gallus*/*Bambusicola*/*Francolinus*) was difficult to reconstruct in many studies, which may be due to a rapid radiation or a limited data. This clade formed a sister group with *Coturnix*/*Alectoris* (Shen et al. 2010), *Pavo* (Jiang et al. 2014), or was rooted at the base of the phasianids (Shen et al. 2014).

In this study, the sample of *F. pintadeanus* was collected from Hainan, China in 2008. The voucher specimen (6680) was deposited in the National Zoological Museum, Institute of Zoology, Chinese Academy of Sciences. The total genomic DNA was extracted from muscle tissue using phenol-chloroform method (Yang et al. 2010). Sequencing was performed using the Illumina Hiseq2000 high-throughput sequencing system of Shenzhen Huada Gene Technology Co. ltd, with the gaps in the assembly filled in by direct sequencing. The length of *F. pintadeanus* mitogenome sequence is 16,693 bp, and has been deposited in GenBank with the accession number KX196445. This mitogenome contains 13 protein-coding genes (nad1-6, nad 4L, cox1-3, cytb, atp6 and atp8), two ribosomal RNA genes (rrnS and rrnL), 22 tRNA genes and one non-coding control region. The base composition of mitogenome is 24.3% T, 31.8% C, 30.4% A and 13.5% G, which shows slightly A + T biased (54.7%). All PCGs are initiated by typical ATG codons except for cox1 (GTG). Nine PCGs harbor the complete termination codon TAN (eight genes with TAA, nad6 with TAG), one gene owns AGG as termination codon (cox1) and the remaining 3 genes (nad2, cox3 and nad4) end with an incomplete termination codon (T). The predicted secondary structure of rrnS contains three domains with 46 stem-loop structures, while rrnL includes six domains with 59 stem-loop structures. All of tRNA genes are shown to be folded into the expected clover-leaf secondary structures except that the trnS(agy) lacks the dihydrouridine (DHU) stem, which is replaced by a simple loop. The control region of F. pintadeanus contains three domains (I-III), the ETAS domain (nt 1-314), conserved central domain (nt 315-784) and CSB domain (nt 785-1 170).

Phylogenetic trees were estimated by maximum likelihood (ML) method using RAxML 7.0.3 (Stamatakis 2006) and Bayesian inference (BI) method using MrBayes 3.2 (Ronquist & Huelsebeck, 2003) under the GTR + I + G model. The ML and BI trees based on a concatenated dataset of 12 protein-coding gene sequences encoded by H-strand (PCG_H) showed that the monophyly of the pheasants and partridges was not supported (Figure 1), which indicated that the Phasianidae birds have experienced a rapid radiation (Jiang et al. 2014). *F. pintadeanus* was well supported as the sister group of the clade containing *Gallus gallus gallus* and *Bambusicola thoracica* (Figure 1). Then, this clade formed a sister group with a clade including *Pavo muticus* and *Argusianus argus* (Figure 1), which was consistent with some previous studies (Jiang et al. 2014, Meiklejohn et al. 2014).

**Figure 1.**
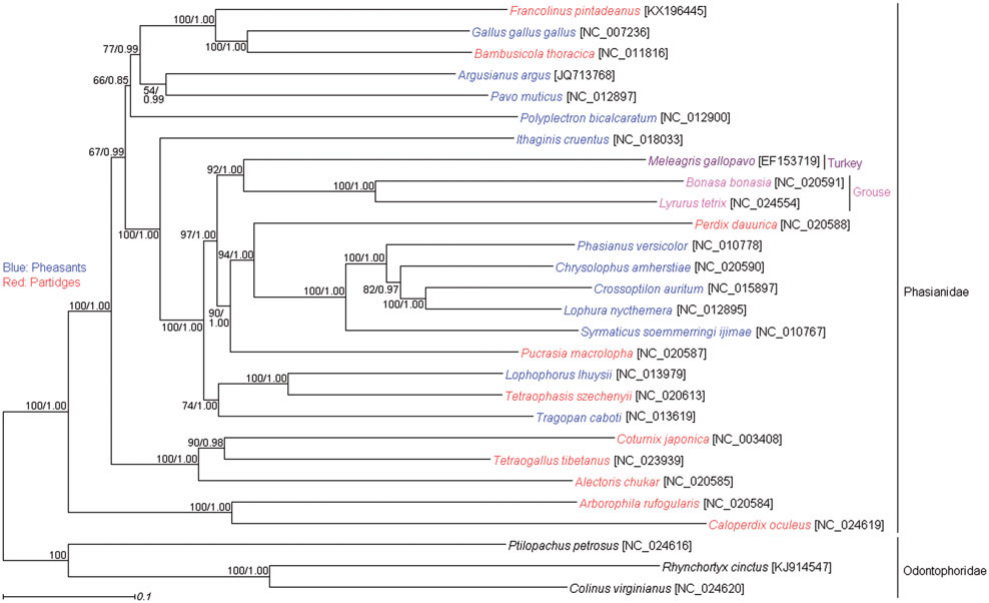
The maximum likelihood (ML) and Bayesian inference (BI) phylogenetic trees based on PCG_H dataset. Numbers on each node correspond to the bootstrap percentage values of ML analysis and the posterior probability values of the BI analysis.
